# 

**Published:** 1910-09

**Authors:** 


*The following donations have been received since the publication of the List in June.*

*August 31st*, 1910.

| | |
|---|---|
| American Urological Association (1) .. .. | 1 volume. |
| Chicago Pathological Society (2) .. .. | 1 ,, |
| L. M. Griffiths (3) .. .. .. .. .. | 7 volumes. |

Unbound periodicals have been received from Dr. James Swain.

### SEVENTY-SEVENTH LIST OF BOOKS.

The titles of books mentioned in previous lists are not repeated.

The figures in brackets refer to the figures after the names of the donors, and show by whom the volumes were presented. The books to which no such figures are attached have either been bought from the Library Fund, or received through the *Journal.*

**Allbutt and H. D. Rolleston, Sir C.** [Eds.] *A System of Medicine.* Vol. VII 1910
**Allen, R. W.** .. .. *Vaccine Therapy* .. .. .. 3rd Ed. 1910
**Althaus, J.** .. .. *On Sclerosis of the Spinal Cord* .. .. (3) 1885
**Bennati, F.** .. .. *Recherches sur le Mécanisme de la Voix humaine* .. .. .. .. .. .. .. (3) 1832
**Bennet, J. H.** .. .. *Nutrition in Health and Disease* (3) 2nd Ed. 1876
**Billington, W.** .. *Movable Kidney* .. .. .. .. .. .. 1910
**Boyce, Sir R. W.** .. *Mosquito or Man* .. .. .. .. 2nd Ed. 1910
**Calkins, G. N.** .. *Protozoölogy* .. .. .. .. .. .. .. .. 1910
**Candy, A. P. Luff and H. C. H.** *A Manual of Chemistry* [4th] Ed. 1910
**Culpeper, [C.]** .. *Pharmacopoeia Londinensis* .. .. .. (3) [1802]
**Fothergill, W. E.** .. *Golden Rules of Obstetric Practice* .. 6th Ed. [1910]
**Haig, A.** .. .. *Uric Acid in the Clinic* .. .. .. .. .. 1910
**Hart, A. H.** .. .. *How to Cut the Drug Bill* .. .. .. 2nd Ed. 1910
**Herman, G. E.** .. *Difficult Labour* .. .. .. .. [5th] Ed. 1910
**Hutchinson, Sir F. Treves and J.** *A Manual of Operative Surgery* Vol. II. 3rd Ed. 1910
**Kelynack, T. N.** [Ed.] *Medical Examination of Schools and Scholars* .. .. .. .. .. .. .. .. .. 1910
**Luff and H. C. H. Candy, A. P.** *A Manual of Chemistry* [4th] Ed. 1910
**Moynihan, B. G. A.** *Gall-Stones* .. .. .. .. .. .. 2nd Ed. 1905
**Pavlov, I. P.** .. .. *The Work of the Digestive Glands* (Trans. by W. H. Thompson) .. .. .. 2nd Ed. 1910
*Portland Hospital, Report of the Committee of the* .. .. .. (3) 1901
**Potts, G. E. Shuttleworth and W. A.** *Mentally Deficient Children* 3rd Ed. 1910
**Rolleston, Sir C. Allbutt and H. D.** [Eds.] *A System of Medicine* Vol. VII. 1910
**Salmon, W.** .. .. *Seplasium : the Compleat English Physician* (3) 1693
**Savill, T. D.** .. .. *A Prescriber's Companion* (Ed. by C. F. Harford .. .. .. .. .. 4th Ed. 1910
**Schofield, A. T.** .. *How to Keep Fit* .. .. .. .. .. .. .. 1910
**Shuttleworth and W. A. Potts, G. E.** *Mentally Deficient Children* 3rd Ed. 1910
**Treves and J. Hutchinson, Sir F.** *A Manual of Operative Surgery* Vol. II. 3rd Ed. 1910
**Turner, W. A.** .. *Three Lectures on Epilepsy* .. .. .. .. 1910
**Walton, A. J.** .. *Fractures and Separated Epiphyses* .. .. 1910
**Walton, H. H.** .. *Operative Ophthalmic Surgery* .. .. (3) 1853
**Whitla, Sir W.** .. *Pharmacy, Materia Medica and Therapeutics* 9th Ed. 1910
**Wingfield, H. E.** .. *An Introduction to Hypnotism* .. .. .. .. 1910
**Winslow, L. F.** .. *The Suggestive Power of Hypnotism* .. .. [1910]

TRANSACTIONS, REPORTS, JOURNALS, &c.

| | | |
|---|---|---|
| American Dermatological Association, Transactions of the .. .. | | 1909 |
| American Journal of Ophthalmology, The .. .. .. | Vol. XXVI | 1909 |
| American Urological Association, Transactions of the | (1) Vol. I | 1907 |
| Annales de Dermatologie et de Syphiligraphie .. .. | Tom. X | 1909 |
| Archiv fur klinische Chirurgie .. .. .. .. .. .. | Bd. XC | 1909 |
| Bristol Medico-Chirurgical Journal, The .. .. .. | Vol. XXVII | 1909 |
| Bulletin de la Socéité française de Dermatologie .. .. .. .. | | 1909 |
| Bulletin de l'Académie royale de Médecine de Belgique | Tom. XXIII | 1909 |
| Chicago Pathological Society, Transactions of the | (2) Vol. VII | 1906–09 |
| Clinical Journal, The .. .. .. .. .. .. .. .. | Vol. XXXV | 1910 |
| Hospitals— | | |
| Johns Hopkins Hospital Reports, The .. .. .. | Vol. XV | 1910 |
| St. Thomas's Hospital Reports .. .. .. | Vol. XXXVII | 1910 |
| Ireland, Transactions of the Royal Academy of Medicine in | Vol. XXVIII | 1910 |
| Journal of Medical Research, The.. .. .. .. .. .. | Vol. XXI | 1909 |
| Journal of the American Medical Association, The .. | Vol. LIII | 1909 |
| Library Association Record, The .. .. .. .. .. | Vol. XI | 1909 |
| Library, The .. .. .. .. .. .. .. .. .. .. .. .. | Vol. X | 1909 |
| Library World, The .. .. .. .. .. .. .. .. | Vol. XI | 1909 |
| Medical Chronicle, The .. .. .. .. .. .. .. | Vol. XVIII | 1909–10 |
| Medical Education in the United States and Canada, Report on .. | | 1910 |
| Ophthalmological Transactions .. .. .. .. | Vol. XXIX | 1909 |
| Parasitology .. .. .. .. .. .. .. .. .. .. .. | Vol. II | 1909 |
| Progressive Medicine .. .. .. .. .. .. .. .. | Vols. I, II | 1910 |
| Public Health .. .. .. .. .. .. .. .. | Vols. XXI, XXII | 1908-09 |
| Rat (The) in relation to the Public Health, Report on .. .. .. | | 1910 |
| Revue de Chirurgie .. .. .. .. .. .. .. .. .. .. | Tom. XL | 1909 |
| Revue de Gynécologie .. .. .. .. .. .. .. .. .. | Tom. XIII | 1909 |
| Revue générale d'Ophtalmologie .. .. .. .. | Tom. XXVIII | 1909 |
| Sleeping Sickness Commission, Reports of the .. .. | No. X | 1910 |

### Local Medical Notes.

**The Pathological Society of Great Britain and Ireland** held its summer meeting at the University on July 8th and 9th. There was a good attendance, and the meeting was most successful.

Friday, the 8th, was devoted to bacteriology, and many problems of practical interest came under discussion. Among these there was an interesting paper by Dr. Martin upon the effect of acids, alkalis and phosphates on the growth of gonococci, and a demonstration of cultures obtained upon a new medium.

Dr. Ainley Walker described some experiments showing the limitations of the carbohydrate tests for streptococci. Professor Sims Woodhead detailed his experiences in purifying waters by the use of chlorine. One part of chlorine in seven million parts of water is sufficient to kill off B.Coli in a few minutes. Dr. Henry described the characteristics of an anærobic streptothrix occurring in a case of cerebro-spinal meningitis, and mentioned some cases of tuberculous meningitis in which there was a polymorphonuclear leucocytosis, even when a secondary infection was not demonstrable. In the discussion following this paper several speakers called attention to the early polymorphonuclear leucocytosis in tuberculous conditions; this has not yet been widely recognised.

Professors Walker Hall and Emrys-Roberts discussed the question of typhoid carriers, illustrating their remarks by a record of the case at present under their observation, together with Dr. D. S. Davies. The effect of drugs and vaccines, etc., upon the output of the bacilli, agglutinins, etc., was shown in charts containing the results of daily observations upon fæces and blood and urine extending over a period of fifteen months.

Saturday was devoted to morbid anatomy and hæmatology. A valuable paper by Professor J. Michell Clarke upon the action of X-rays in leukæmia evoked considerable discussion, and led to a proposal for the simplification of the terminology used by hæmatologists. At a later stage the manufacture of new terms was again referred to, and a suggestion was made that some definite steps be taken to obtain a standard nomenclature. Nothing definite came of the matter, but the tenour of the speeches indicated the probability of such a move in the near future.

Dr. Price Jones placed on record a number of blood counts showing the errors occasioned by the use of Toisson's fluid as a diluting agent, and advocated the more general use of Hayem's fluid. A feature of the meeting was the contribution of Dr. Alexander Bruce upon the ætiology of disseminated sclerosis. He showed a large number of excellent photo-micrographs illustrating the spread of fibrous tissue around and along veins and lymphatics. Some eighty microscopes were used for the demonstrations, and a number of naked-eye specimens were also shown. Dr. Muir exhibited a new electrical warm stage, and Mr. Lechmere demonstrated the results of his work upon cider yeasts.

There were other papers of interest, but we mention these as being of present interest to the general reader.

On Friday evening the Society held its usual dinner at the Clifton Down Hotel, and on Saturday the combined staffs of the Royal Infirmary and General Hospital entertained the members at lunch at the Queen's Hotel. The University and Literary Club made the members of the Society honorary members during the meeting, and many availed themselves of the privileges offered.

**University of Bristol.**—FACULTY OF MEDICINE:—

EXAMINATION SUCCESSES.

M.B., B.Ch. BRISTOL.—C. W. Brasher, A. L. Flemming, L. A. Moore, L. N. Morris, F. C. Nicholls, V. B. Green-Armytage, J. W. Taylor, W. W. King. *Part I only:* S. H. Kingston

*Intermediate M.B.:* B. G. Derry, A. G. Hieber. *Second Examination for B.D.S.* (*Dental*)*:* R. H. Basker, J. W. Gilbert, E. J. Handford.

M.D. Lond.—C. E. K. Herapath, L. J. Short.

University of London.—*First Examination for Medical Degrees:* A. W. Adams, F. K. Hayman, G. M. Jackson, Mary E. Joll, D. G. C. Tasker. *Second Examination, Part I:* H. J. McCurrich, H. J. D. Smythe. *Part II:* F. W. T. Clemens, A. H. White.

Conjoint Board of London.—*Chemistry and Physics:* P. Smith, E. W. Thomas. *Physics only:* W. A. Culverwell. *Biology:* G. S. Terry. *Pharmacy:* R. G. Johnson, J. W. Gilbert. *Anatomy and Physiology:* L. Page. *Surgery:* W. W. Pratt, R. C. Clarke,* A. J. Wigmore,* B. C. Eskell,* R. M. Hiley,* W. C. Goodden.

Indian Medical Service.—*Promotion—Lieutenant to Captain:* V. B. Green-Armytage, A. N. Thomas, F. Shingleton Smith.

### APPOINTMENTS.

**Leicester Infirmary.**—C. A. Joll, M.B., B.S., F.R.C.S., L.D.S., has been appointed House Surgeon.

**Cardiff University College; Cardiff Infirmary.**—E. Emrys-Roberts, M.D. Liverpool, has been appointed Professor of Pathology at University College, and Pathologist to the Infirmary.

---

**Editorial Secretary, change of address.**—Dr. J. A. Nixon is now at 19 Mortimer Road, Clifton.

* Denotes qualification.

